# Prediction of genomic breeding values using new computing strategies for the implementation of MixP

**DOI:** 10.1038/s41598-017-17366-2

**Published:** 2017-12-08

**Authors:** Linsong Dong, Ming Fang, Zhiyong Wang

**Affiliations:** 1 Key Laboratory of Healthy Mariculture for the East China Sea, Ministry of Agriculture; Fisheries College, Jimei University, Xiamen, Fujian P.R. China; 2 Laboratory for Marine Fisheries Science and Food Production Processes, Qingdao National Laboratory for Marine Science and Technology, Qingdao, 266235 P.R. China

## Abstract

MixP is an implementation that uses the Pareto principle to perform genomic prediction. This study was designed to develop two new computing strategies: one strategy for nonMCMC-based MixP (FMixP), and the other one for MCMC-based MixP (MMixP). The difference is that MMixP can estimate variances of SNP effects and the probability that a SNP has a large variance, but FMixP cannot. Simulated data from an international workshop and real data on large yellow croaker were used as the materials for the study. Four Bayesian methods, BayesA, BayesCπ, MMixP and FMixP, were used to compare the predictive results. The results show that BayesCπ, MMixP and FMixP perform better than BayesA for the simulated data, but all methods have very similar predictive abilities for the large yellow croaker. However, FMixP is computationally significantly faster than the MCMC-based methods. Our research may have a potential for the future applications in genomic prediction.

## Introduction

The advent of next generation sequencing technology has accelerated the development of the theory behind quantitative molecular genetics approaches, such as quantitative trait loci (QTL) mapping, genome-wide association (GWA) studies and genomic selection. Genomic selection was first proposed by Meuwissen *et al*. as an efficient method to predict animal breeding outcomes^1^. Recently, various implementations have been proposed for genomic prediction, such as genomic best linear unbiased prediction (GBLUP)^2^, ridge-regression BLUP (RRBLUP), BayesA, BayesB^1^, BayesCπ^3^, BayesLASSO^4,5^, BayesSSVS^6^, fast Bayesian methods^7,8^, MixP^9^, among others. GBLUP and RRBLUP assume a constant variance for all SNP loci, which may be an imprecise assumption if a trait is affected by a small number of QTL loci^1^. Bayesian methods propose more flexible prior assumptions for SNP effects (or variances). Generally, the prior distributions of Bayesian methods assume that there are large variances in some SNP loci and small or even zero variances at other loci, which seems to be more realistic. The implementations of Bayesian methods, such as BayesA, B, Cπ and LASSO, are mainly based on Markov chain Monte Carlo (MCMC) algorithms, requiring much more computation time to estimate SNP effects. To increase the computational speed, researchers have suggested some fast Bayesian methods, such as fast BayesB^7^ and emBayesB^8^. Yu and Meuwissen proposed another fast Bayesian method using the Pareto principle to perform genomic prediction^9^.

The Pareto principle was proposed by the economist Vilfredo Pareto at the beginning of the 20th century^10^. This principle states that approximately 20% of the population possesses 80% of the wealth in a country. Similar theories have been further applied in various fields, such as in genomic prediction by Yu and Meuwissen^9^, resulting in the method termed MixP. The prior distribution of MixP is a mixture of two normal distributions, which assumes that *x*% of the SNPs cause (100 − *x*)% of the genetic variance, so the remaining (100 − *x*)% of SNPs decide the remaining *x*% of genetic variance. Here we assume *γ* = *x*%, and (1 − *γ*) = (100 − *x*)%. The large and small variances are proposed as follows^9^:

1 $$\{\begin{array}{c}{{\sigma }_{1}}^{2}=\frac{(1-\gamma ){V}_{g}}{\gamma M}\\ {{\sigma }_{2}}^{2}=\frac{\gamma {V}_{g}}{(1-\gamma )M}\end{array},$$

where *σ*_1_^2^ and *σ*_2_^2^ represent the large and small variance of a SNP effect, respectively; *V*_*g*_ is the total additive genetic variance; *M* is the number of SNPs; and *γ* ≤ 0.5. The prior for MixP assumes that all SNPs have effects, but each SNP has only two possible variances: *σ*_1_^2^ or *σ*_2_^2^. This is similar but not completely identical to the assumptions found in two other Bayesian methods (BayesA and BayesCπ). In BayesA, the prior also assumes that all SNPs have effects but each SNP has its own variance. The variances of the SNP effects in BayesA follow an inverse-chi-squared distribution^1,11^. The prior for BayesCπ assumes that SNPs with non-zero effects have a common variance, which is similar to the assumption of MixP, which assumes that “large” SNPs have a common variance (*σ*_1_^2^). However, SNP effects with small variance may be shrunk to zero in BayesCπ.

MixP is also a fast Bayesian method that is not based on a MCMC algorithm^9^. However, a multivariate normal density and an inverse matrix are included in the derivation, increasing the difficulty in understanding the derivation. In the nonMCMC-based MixP, the *γ* is given but not estimated, such that the optimal value of *γ* should be searched using a cross-validation. However, the parameter *γ* can be estimated using the MCMC algorithm. For the sake of convenience in distinguishing different algorithms, the MixP not based on the MCMC algorithm is termed fast MixP (FMixP), and the MixP based on the MCMC algorithm is termed MCMC-based MixP (MMixP) here.

In this study, we developed two new computing strategies for FMixP and MMixP, respectively. The first strategy used a product of univariate densities instead of the multivariate normal density to estimate SNP effects for FMixP; the second strategy attempted to use the MCMC algorithm to derive the solutions for MMixP. In addition, the strategies were used to analyse the results on simulated data from an international workshop and real data on large yellow croaker, and compared the predictive abilities with estimations by BayesA and BayesCπ.

## Results

### Results for simulated data

**Figure 1:**
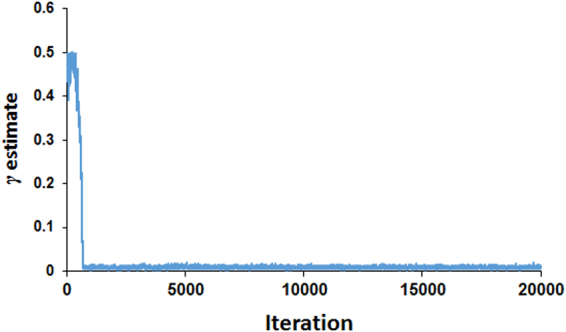
γ estimates in the Gibbs sampling cycles of MMixP in simulated data.

The predictive results of various Bayesian methods for the simulated data are shown in Table 1. The predictive accuracies are very close in BayesCπ, MMixP, and FMixP (*γ* = 0.07). The accuracy of BayesA is lower than that of BayesCπ, MMixP, and FMixP (*γ* = 0.07), but higher than FMixP when *γ* = 0.5. BayesCπ and MMixP yield comparatively accurate estimates for *π* and *γ*, respectively. As there are 48 QTLs simulated in the genome, the true value of *π* (or *γ*) is 48/5726 ≈ 0.0084, which is very close to the values estimated by BayesCπ and MMixP. The *γ* estimates in the Gibbs sampling cycles are shown in Fig. 1. We can find that the value converges when the Gibbs sampling runs at ~1000th cycle.

**Table 1:** Correlation and regression coefficients of TBV on GEBV for various methods in simulated data.

	*r*_(TBV_,_GEBV)_	*b*_(TBV_,_GEBV)_
BayesA	0.807	0.850
BayesCπ	0.885 (^a^ *π* = 0.0096)	0.896
^b^FMixP	0.882 (*γ* = 0.07)	0.980 (*γ* = 0.07)
FMixP (*γ* = 0.5)	0.753	0.851
MMixP	0.885 (^c^ *γ* = 0.0092)	0.893

^a^ *π* is the probability of a SNP with non-zero effect estimated by BayesCπ.

^b^The optimized result estimated by FMixP when *γ* equals the value in the parentheses.

^c^ *γ* is the probability of a SNP with large variance estilmated by MMixP.

*r*_(TBV,GEBV)_ and *b*_(TBV,GEBV)_ represent the correlation and regression coefficients of TBV on GEBV, respectively.

**Figure 2:**
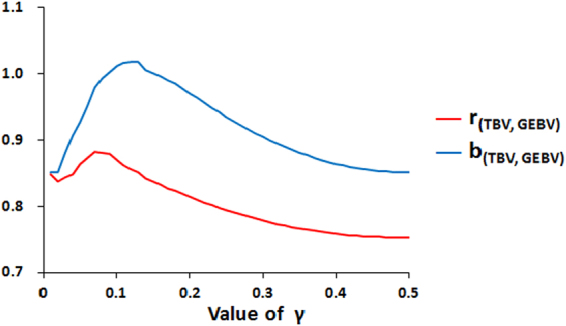
Graphs of the correlation and regression coefficients of TBV on GEBV for FMixP against *γ* in simulated data.

**Figure 3:**
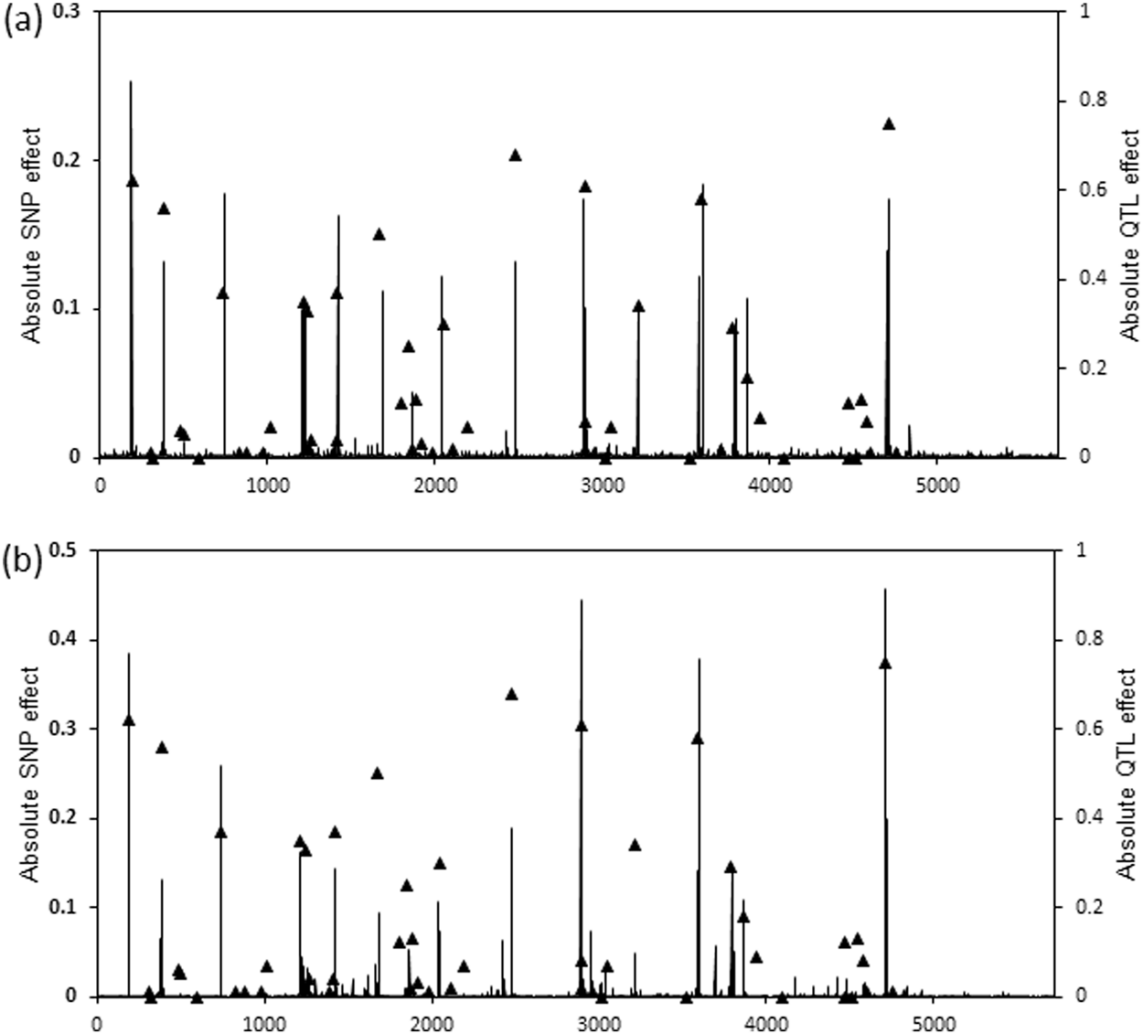
Distributions of absolute SNP effects estimated by FMixP and MMixP in simulated data. (**a**) FMixP with *γ* = 0.07; (**b**) MMixP. ▲ represents the location and effect of QTL in genome.

We compared the predictive results between MixP introduced by Yu and Meuwissen^9^ and our FMixP, and found that the two derivations could yield the same prediction accuracies. Graphs of the correlation and regression coefficients of TBV on GEBV (*r*_(TBV_,_GEBV)_ and *b*_(TBV_,_GEBV)_, respectively) against *γ* for FMixP are presented in Fig. 2. Both measures of accuracy follow a similar trend in response to *γ*. Overall, FMixP yields the highest accuracy when the value of *γ* is close to 0.07, but this value is higher than the true value (0.0084). The distributions of SNP effects estimated by FMixP and MMixP are shown in Fig. 3. All the QTLs with absolute effects >0.2 can be located by the nearby SNPs in both methods, indicating that the MixP may be a promising implementation in GWA study.

### Results for real data

**Figure 4:**
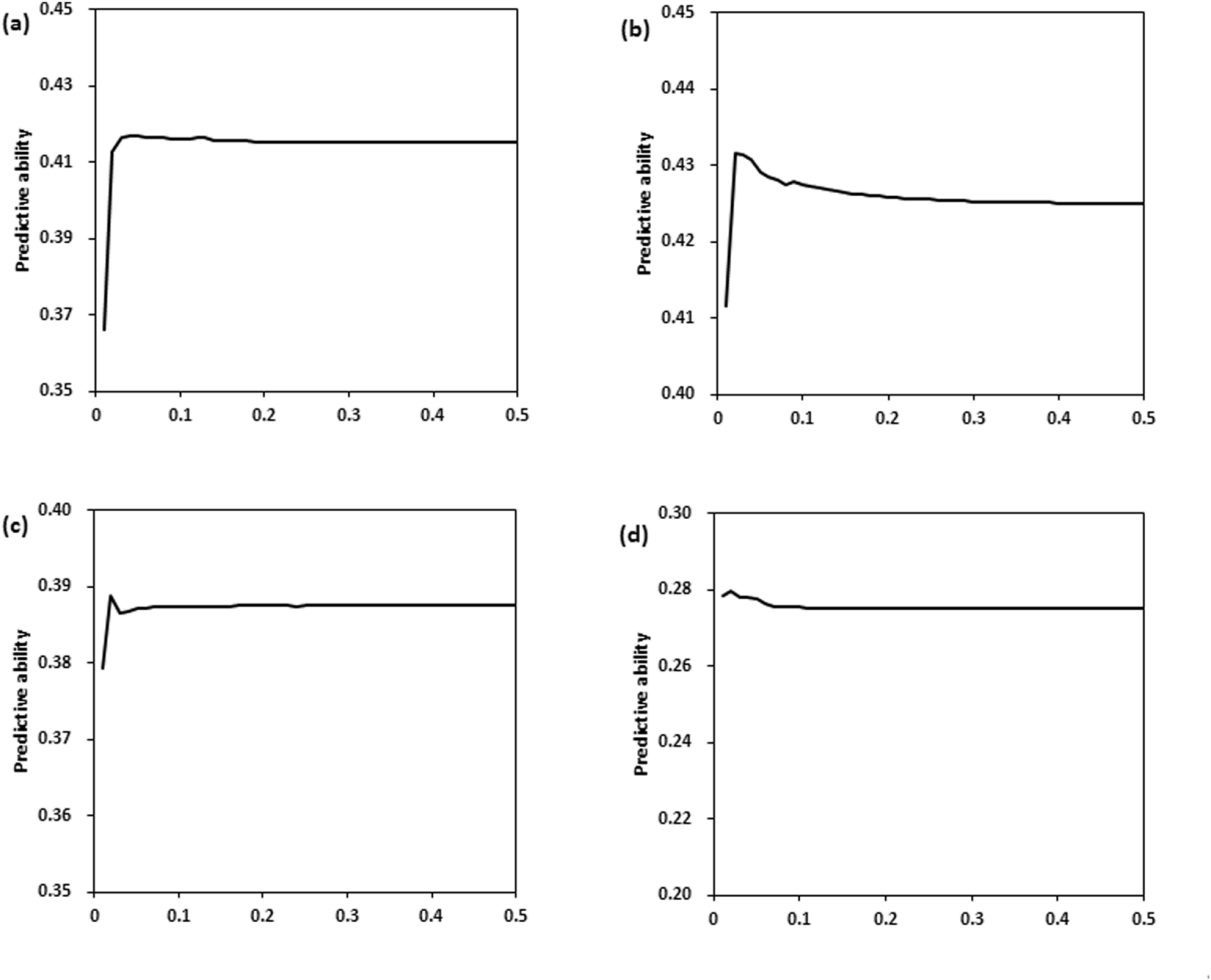
Graphs of the predictive ability of FMixP against *γ* for four traits in large yellow croaker. (**a**) Body weight; (**b**) Body length; (**c**) Body height; (**d**) Length/height.

Table 2 shows the predictive abilities of various Bayesian methods for four quantitative traits in large yellow croaker. The results estimated by BayesA, BayesCπ, MMixP and FMixP are very similar for all traits, with no within-trait difference in predictive ability greater than 0.01. The value of *γ* (the probability of a SNP with a large variance) estimated by MMixP is much higher than that estimated in the simulated data, indicating that there may be many QTLs affecting the phenotypes. The results of FMixP show that predictive abilities are optimized when the probability of a SNP with a large variance in specific traits is 0.02 or 0.05. However, these optimal points are not obvious because the predictive abilities are still very close to the best results when *γ* = 0.5, which is not consistent with the results from the simulated data. Figure 4 shows graphs of the predictive ability against *γ* for FMixP for various traits. It shows that the value of *γ* barely affects the predictive ability as long as *γ* is larger than 0.05 or even 0.02. The values of *γ* estimated by MMixP are 0.28, 0.32, 0.27 and 0.31 for the traits body weight, body length, body height and length/height, respectively.

**Table 2:** Predictive abilities of various methods for four traits in large yellow croaker.

Trait	Predictive ability (Mean ± SE)				
	BayesA	BayesCπ	^a^FMixP	FMixP (*γ* = 0.5)	MMixP
Body weight	0.413 ± 0.040	0.413 ± 0.040	0.417 ± 0.041 (*γ* = 0.05)	0.415 ± 0.040	0.412 ± 0.040
Body length	0.431 ± 0.033	0.430 ± 0.033	0.432 ± 0.032 (*γ* = 0.02)	0.425 ± 0.033	0.432 ± 0.033
Body height	0.388 ± 0.037	0.388 ± 0.037	0.389 ± 0.038 (*γ* = 0.02)	0.387 ± 0.037	0.387 ± 0.037
Length/height	0.274 ± 0.038	0.273 ± 0.038	0.278 ± 0.036 (*γ* = 0.05)	0.275 ± 0.037	0.277 ± 0.038

^a^The optimized result estimated by FMixP when *γ* equals the value in the parentheses.

### Computation time

Table 3 shows the computation time of each method for the simulated data and the trait length/height in large yellow croaker. The Fortran90 codes were run in a computer with an Intel Xeon CPU E7-4820. The computation time of MMixP is the longest in all statistical methods. Compared with the BayesCπ, the computational speed of BayesA is slightly slower in the simulated data but slightly faster in the real data. However, all MCMC-based Bayesian methods show a much slower computational speed than FMixP. The computation time for FMixP with *γ* = 0.5 is longer than that for FMixP with *γ* = 0.05 in the simulated data, but this difference is not obvious in the real data. We also compared the computation time between MixP introduced by Yu and Meuwissen and our FMixP, and the results showed that the time of their MixP was approximately 20~25% longer than that of our FMixP.

**Table 3:** Computation time of genomic prediction using various Bayesian methods for trait length/height.

Computation time (minute)	BayesA	BayesCπ	MMixP	FMixP (*γ* = 0.05)	FMixP (*γ* = 0.5)
Simulated data	309.6	268.1	428.7	0.48	1.8
length/height	210.5	229.2	263.1	0.02	0.02

## Discussion

In this study, we compared the predictive abilities among BayesA, BayesCπ, MMixP and FMixP. When *γ* = 0.5, the results of FMixP are equivalent to those of GBLUP or RRBLUP, an observation which was also mentioned by Yu and Meuwissen^9^. Hence, the predictive result of FMixP when *γ* = 0.5 is the same as that of GBLUP in Shepherd *et al*.^8^, in which the same simulated data was used. Therefore, we actually compared the results of five methods (i.e., BayesA, BayesCπ, MMixP, FMixP and GBLUP) in this study. The results show that the ranking of the predictive results among the different methods is not consistent between the simulated and real data. In the simulated data, the ranking according to predictive accuracy is: BayesCπ ≈ MMixP ≈ FMixP (*γ* = 0.07) > BayesA > GBLUP. However, all of the methods yield almost the same result within a given trait in real data from large yellow croaker. A reasonable explanation may be that there is a small number of QTLs in the simulated data but many more QTLs in the real data. There are two reasons that support this speculation: (i) The simulated results of Yu and Meuwissen showed that accuracy was not sensitive to *γ* when the number of QTL loci was large, but FMixP with *γ* < 0.5 performed better than GBLUP if there was a small number of QTLs^9^. The results shown in Figs 2 and 4 are consistent with the above two cases. (ii) The values of *γ* estimated by MMixP in simulated data are much lower than that estimated in the real data, indicating there may be many QTL loci affecting the phenotypes in large yellow croaker. Another explanation is that when the LD between markers is not strong, the accuracy may be due to the relationships captured by markers^12,13^. In this case, the GBLUP and various Bayesian methods may yield similar predictive results.

In addition to the predictive accuracy, computational speed is another important aspect in genomic prediction. This study shows that FMixP is significantly faster than the MCMC-based Bayesian methods. The main reason for this difference is that FMixP is not based on MCMC algorithms which are sampling processes and require many cycles to obtain a precise solution. It shows that the computation time for BayesCπ is slightly longer than BayesA in the real data, but slightly shorter in the simulated data. This is because the computational speed of BayesCπ is based on the value of *π*. A smaller *π* means more SNPs have zero effects and thus do not need to be sampled from the posterior normal distribution. MMixP needs more computation time than BayesA and BayesCπ, because there are more variables that need to be sampled in MMixP. For example, the SNP effect with variance equalling zero is not sampled in BayesCπ. However, all SNP effects need to be sampled in each Gibbs sampling cycle in MMixP, because each SNP may have a large or small variance. The computational speed for FMixP with *γ* = 0.05 is faster than for FMixP with *γ* = 0.5 in the simulated data. The possible reason for this is that the number of QTLs is very small in the simulated data. FMixP with *γ* = 0.05 is closer to the real QTL distribution, so that FMixP with *γ* = 0.05 has a faster convergence speed. This also suggests that there may be more QTL loci in the real data, because there is no obvious difference in computation time for FMixP with *γ* = 0.05 or 0.5.

This study proposed two new computing strategies: one strategy for FMixP and the other one for MMixP. Compared with the derivation of Yu and Meuwissen^9^, we used a simpler derivation to obtain the solutions in FMixP. The advantage of FMixP is the extremely fast computational speed. However, the probability of a SNP having a large variance (represented as *γ*) and variances of SNP effects cannot be estimated by this implementation. Instead, using the MCMC algorithm can estimate the *γ* and various variances, but the computational speed is significantly slower than FMixP. The two strategies may provide some references to others who want to perform genomic prediction in the future.

## Material and Methods

### Ethics approval

This study and all experimental protocols were approved by the Animal Care and Use Committee of the Fisheries College of Jimei University (Animal Ethics no. 1067). All methods were performed in accordance with approved guidelines.

### Analytical derivation for FMixP

The linear model for genomic prediction was as follows:

2 $${\bf{y}}={\bf{X}}{\bf{u}}+{\bf{B}}{\bf{g}}+{\bf{e}},$$

where **y** is a vector of phenotypic records, **X** is the design matrix for fixed effects, and **u** is a vector of fixed effects. In the simulated data, **X** = (1 1 … 1)′ and **u** is overall mean, whereas in the real data, the fixed effects were the sexual effects, **X**_*i*_ = (1 0) for male and (0 1) for female. **B** is the matrix of SNP genotypes (coded as 0 for genotype ‘*A*_*A*’, 1 for ‘*A*_*a*’ and 2 for ‘*a*_*a*’), **g** is a vector of SNP effects, and **e** is a vector of residual effects, where **e** ~ *N*(0, **I** *σ*_*e*_^2^). Genotypic codes were standardised using the formula: $B{^{\prime} }_{ij}=({B}_{ij}-2{p}_{j})/\sqrt{2{p}_{j}(1-{p}_{j})}$, where *p*_*j*_ is the frequency of allele ‘*a*’ at locus *j*.

In this study, the prior distribution was the same as that described by Yu and Meuwissen^9^. According to the prior distribution for SNP variance, the prior for SNP effect *g*_*j*_ can be written as a mixture of normal distributions:

3 $$\pi ({g}_{j})=\gamma {\varphi }({g}_{j}|0,{{\sigma }_{1}}^{2})+(1-\gamma ){\varphi }({g}_{j}|0,{{\sigma }_{2}}^{2}),$$

where *g*_*j*_ is the effect of SNP *j*.

Here, we used an Iterative Conditional Expectation (ICE) algorithm^7^ to estimate the SNP effects. This algorithm estimates *E*(**g**|**y**) for each SNP effect in turn, where the current effects of the other SNPs are assumed to be known values. For example, if *E*(*g*_*j*_|**y**_*−j*_) is estimated, the current effects of all other SNPs are used to calculate the **y**_*−j*_, i.e.,

4 $${{\bf{y}}}_{-j}={\bf{y}}-{\bf{X}}{\bf{u}}-\sum _{k\ne j}{{\bf{B}}}_{k}{g}_{k},$$

where **B**_*k*_ is a vector from the *k* ^th^ column of **B**. The expectation of SNP effect, *E*(*g*_*j*_|**y**_*−j*_), is estimated by a Bayesian model^7,9^:

5 $$\begin{array}{rcl}E({g}_{j}|{{\bf{y}}}_{-j}) & = & {\int }_{-\infty }^{+\infty }{g}_{j}f({g}_{j}|{{\bf{y}}}_{-j})d{g}_{j}\\  & = & \frac{{\int }_{-\infty }^{+\infty }{g}_{j}f({{\bf{y}}}_{-j}|{{\bf{B}}}_{j}{g}_{j},{\bf{I}}{{\sigma }_{e}}^{2})\pi ({g}_{j})d{g}_{j}}{{\int }_{-\infty }^{+\infty }f({{\bf{y}}}_{-j}|{{\bf{B}}}_{j}{g}_{j},{\bf{I}}{{\sigma }_{e}}^{2})\pi ({g}_{j})d{g}_{j}}\end{array},$$

where the *f*(**y**_*−j*_|**B**_*j*_ *g*_*j*_, **I** *σ*_*e*_^2^) is a multivariate normal density. Evaluating this multivariate density will be computationally intense because it involves calculating the determinant and inverse of variance-covariance matrix for the data **y**_*−j*_. However, the *f*(**y**_*−*j_|**B**_*j*_ *g*_*j*_, **I** ${\sigma }_{e}^{2}$) is proportional to the product of univariate normal densities *f*(*Y*|*g*_*j*_, *σ* ^2^), where *Y* = (**B**_*j*_′**B**_*j*_)^−1^ **B**_*j*_′**y**_*−j*_ and *σ* ^2^ = (**B**_*j*_′**B**_*j*_)^−1^ ${\sigma }_{e}^{2}$ (See Appendix 2 of Meuwissen *et al*.^7^). Unlike the derivation of Yu and Meuwissen^9^, we did not calculate the multivariate likelihood but simplified the derivation using *f*(*Y*|*g*_*j*_, *σ* ^2^) to replace *f*(**y**_*−j*_|**B**_*j*_ *g*_*j*_, **I** ${{\sigma }_{e}}^{2}$). Thus, the equation (5) can be rewritten as:

6 $$E({g}_{j}|{{\bf{y}}}_{-j})=\frac{{\int }_{-\infty }^{+\infty }{g}_{j}\,f(Y|{g}_{j},{\sigma }^{2})\pi ({g}_{j})d{g}_{j}}{{\int }_{-\infty }^{+\infty }f(Y|{g}_{j},{\sigma }^{2})\pi ({g}_{j})d{g}_{j}}.$$

Combined with equation (3), the numerator of equation (6) can be split into two terms:

7 $$\gamma {\int }_{-\infty }^{+\infty }{g}_{j}f(Y|{g}_{j},{\sigma }^{2}){\varphi }({g}_{j}|0,{\sigma }_{1}^{2})d{g}_{j}+(1-\gamma ){\int }_{-\infty }^{+\infty }{g}_{j}f(Y|{g}_{j},{\sigma }^{2}){\varphi }({g}_{j}|0,{\sigma }_{2}^{2})d{g}_{j}.$$

The first term in formula (7) can be derived as follows:

8 $$\begin{array}{ll} & \gamma {\int }_{-\infty }^{+\infty }{g}_{j}f(Y|{g}_{j},{\sigma }^{2}){\varphi }({g}_{j}|0,{\sigma }_{1}^{2})d{g}_{j}\\ = & \frac{\gamma }{\sqrt{2\pi }}{\int }_{-\infty }^{+\infty }\frac{{g}_{j}}{\sqrt{2\pi }\sigma {\sigma }_{1}}\exp [-\frac{{(Y-{g}_{j})}^{2}}{2{\sigma }^{2}}-\frac{{{g}_{j}}^{2}}{2{\sigma }_{1}^{2}}]d{g}_{j}\\ = & \frac{\gamma }{\sqrt{2\pi }}\exp [-\frac{{Y}^{2}}{2({\sigma }_{1}^{2}+{\sigma }^{2})}]\frac{1}{\sqrt{{\sigma }_{1}^{2}+{\sigma }^{2}}}{\int }_{-\infty }^{+\infty }\frac{{g}_{j}}{\sqrt{2\pi }\frac{\sigma {\sigma }_{1}}{\sqrt{{\sigma }_{1}^{2}+{\sigma }^{2}}}}\exp [-\frac{{({g}_{j}-\frac{Y{\sigma }_{1}^{2}}{{\sigma }_{1}^{2}+{\sigma }^{2}})}^{2}}{2{(\frac{\sigma {\sigma }_{1}}{\sqrt{{\sigma }_{1}^{2}+{\sigma }^{2}}})}^{2}}]d{g}_{j}.\end{array}$$

The last term in formula (8) can be taken as calculating the expected value of *g*_*j*_ in the normal distribution with a mean *Yσ*_1_^2^/(*σ*_1_^2^ + *σ* ^2^) and variance *σ* ^2^ *σ*_1_^2^/(*σ*_1_^2^ + *σ* ^2^), so this term equals *Yσ*_1_^2^/(*σ*_1_^2^ + *σ* ^2^). Thus, the first term of formula (7) becomes:

9 $$\frac{\gamma }{\sqrt{2\pi }}\exp [-\frac{{Y}^{2}}{2({\sigma }_{1}^{2}+{\sigma }^{2})}]\frac{1}{\sqrt{{\sigma }_{1}^{2}+{\sigma }^{2}}}\frac{Y{{\sigma }_{1}}^{2}}{{\sigma }_{1}^{2}+{\sigma }^{2}}.$$

Similarly, the second term becomes:

10 $$\frac{1-\gamma }{\sqrt{2\pi }}\exp [-\frac{{Y}^{2}}{2({\sigma }_{2}^{2}+{\sigma }^{2})}]\frac{1}{\sqrt{{{\sigma }_{2}}^{2}+{\sigma }^{2}}}\frac{Y{\sigma }_{2}^{2}}{{\sigma }_{2}^{2}+{\sigma }^{2}}.$$

Thus, the numerator of equation (6) equals:

11 $$\frac{\gamma }{\sqrt{2\pi }}\exp [-\frac{{Y}^{2}}{2({\sigma }_{1}^{2}+{\sigma }^{2})}]\frac{1}{\sqrt{{\sigma }_{1}^{2}+{\sigma }^{2}}}\frac{Y{\sigma }_{1}^{2}}{{\sigma }_{1}^{2}+{\sigma }^{2}}+\frac{1-\gamma }{\sqrt{2\pi }}\exp [-\frac{{Y}^{2}}{2({\sigma }_{2}^{2}+{\sigma }^{2})}]\frac{1}{\sqrt{{\sigma }_{2}^{2}+{\sigma }^{2}}}\frac{Y{\sigma }_{2}^{2}}{{\sigma }_{2}^{2}+{\sigma }^{2}}.$$

The derivation of the denominator in equation (6) is very similar to that of the numerator, but there is no *g*_*j*_ in the integrand. Therefore, the integral is not to calculate the expected value, but rather to calculate the cumulative probability from −∞ to ∞, so this value is 1. Thus, the denominator in equation (6) can be written as:

12 $$\frac{\gamma }{\sqrt{2\pi }}\exp [-\frac{{Y}^{2}}{2({\sigma }_{1}^{2}+{\sigma }^{2})}]\frac{1}{\sqrt{{\sigma }_{1}^{2}+{\sigma }^{2}}}+\frac{1-\gamma }{\sqrt{2\pi }}\exp [-\frac{{Y}^{2}}{2({\sigma }_{2}^{2}+{\sigma }^{2})}]\frac{1}{\sqrt{{\sigma }_{2}^{2}+{\sigma }^{2}}}.$$

Thus, we derive the final form for equation (6),

13 $$E({g}_{j}|{{\bf{y}}}_{-j})=\frac{\gamma \frac{Y{{\sigma }_{1}}^{2}}{{{\sigma }_{1}}^{2}+{\sigma }^{2}}+(1-\gamma )\exp [\frac{1}{2}(\frac{{Y}^{2}}{{{\sigma }_{1}}^{2}+{\sigma }^{2}}-\frac{{Y}^{2}}{{{\sigma }_{2}}^{2}+{\sigma }^{2}})]\frac{\sqrt{{{\sigma }_{1}}^{2}+{\sigma }^{2}}}{\sqrt{{{\sigma }_{2}}^{2}+{\sigma }^{2}}}\frac{Y{{\sigma }_{2}}^{2}}{{{\sigma }_{2}}^{2}+{\sigma }^{2}}}{\gamma +(1-\gamma )\exp [\frac{1}{2}(\frac{{Y}^{2}}{{{\sigma }_{1}}^{2}+{\sigma }^{2}}-\frac{{Y}^{2}}{{{\sigma }_{2}}^{2}+{\sigma }^{2}})]\frac{\sqrt{{{\sigma }_{1}}^{2}+{\sigma }^{2}}}{\sqrt{{{\sigma }_{2}}^{2}+{\sigma }^{2}}}}.$$

The fixed effects are estimated in each iteration by the formula: û  = (**X**′**X**)^−1^ **X**′(**y**−**Bĝ**). We judged the convergence of solutions at the *t*th iteration according to the formula (**G** ^*t*^−**G** ^*t*−1^)′(**G** ^*t*^−**G** ^*t*−1^)/(**G** ^*t*^′**G** ^*t*^) < 10^−8^, where **G** = (** û**′ **ĝ**′)′.

### Derivation for MMixP

FMixP does not estimate the parameter *γ*, such that a direct search should be used to obtain the optimal value of *γ* in genomic prediction. However, the value of *γ* can be estimated by the MCMC algorithm. With MMixP, the prior distributions of various variables, such as *γ*, **u**, **g**, ${{\sigma }_{1}}^{2}$, ${{\sigma }_{2}}^{2}$ and ${{\sigma }_{e}}^{2}$, are required. The priors for *γ*, **u** and *σ*_*e*_^2^ were assumed to follow uniform distributions. The prior for *g*_*j*_ depended on the *γ* and variances:

14 $${g}_{j}|\gamma ,{\sigma }^{2} \sim \{\begin{array}{c}N(0,{\sigma }_{1}^{2})\,\,{\rm{with}}\,{\rm{probability}}\,\gamma \\ N(0,{\sigma }_{2}^{2})\,\,{\rm{with}}\,{\rm{probability}}\,(1-\gamma )\end{array},$$

where *γ* is the probability that a SNP has a large variance, and ${{\sigma }_{1}}^{2}$ and ${{\sigma }_{2}}^{2}$ represent the large and small variance, respectively. The priors of ${{\sigma }_{1}}^{2}$ and ${{\sigma }_{2}}^{2}$ were assumed to follow the inverse-chi-squared distributions:

15 $$\{\begin{array}{c}{\sigma }_{1}^{2} \sim {\chi }^{-2}(v,{s}_{1}^{2})\quad {\rm{where}}\,\,{{s}_{1}}^{2}=\frac{(v-2)(1-\gamma ){V}_{g}}{v\gamma M}\\ {\sigma }_{2}^{2} \sim {\chi }^{-2}(v,{s}_{2}^{2})\quad {\rm{where}}\,\,{{s}_{2}}^{2}=\frac{(v-2)\gamma {V}_{g}}{v(1-\gamma )M}\end{array},$$

The scale parameter *s*_1_^2^ was set because $E({\sigma }_{1}^{2})=\frac{v{s}_{1}^{2}}{v-2}=\frac{(1-\gamma ){V}_{g}}{\gamma M}$ according to the properties of inverse-chi-squared distribution and Pareto principle. A similar method was used to set the parameter *s*_2_^2^. An indicator variable *δ*_*j*_ was used to indicate whether SNP *j* had a large or small variance. The prior for *δ*_*j*_ was $p({\delta }_{j}|\gamma )={\gamma }^{{\delta }_{j}}{(1-\gamma )}^{(1-{\delta }_{j})}$, where *δ*_*j*_ = 1 and *δ*_*j*_ = 0 represent the *σ*_*j*_^2^ = *σ*_1_^2^ with probability *γ* and ${\sigma }_{j}^{2}$ = ${\sigma }_{2}^{2}$ with probability (1 − *γ*), respectively.

The *δ*_*j*_ and g_*j*_ are sampled from their joint conditional distribution, because the sampling strategy of g_*j*_ is dependent on the value of *δ*_*j*_. The joint conditional distribution can be written as:

16 $$f({\delta }_{j},{g}_{j}|{\bf{y}},{\bf{u}},{{\bf{g}}}_{-j},{{\boldsymbol{\delta }}}_{-j},{{\boldsymbol{\sigma }}}^{2},{{\sigma }_{e}}^{2},\gamma )=f({\delta }_{j}|{\bf{y}},{\bf{u}},{{\bf{g}}}_{-j},{{\boldsymbol{\delta }}}_{-j},{{\boldsymbol{\sigma }}}^{2},{{\sigma }_{e}}^{2},\gamma )f({g}_{j}|{\delta }_{j},{\bf{y}},{\bf{u}},{{\bf{g}}}_{-j},{{\boldsymbol{\delta }}}_{-j},{{\boldsymbol{\sigma }}}^{2},{{\sigma }_{e}}^{2},\gamma ),$$

where **g**_*−j*_ and **δ**_*−j*_ represent the vectors of SNP effects and indicator variables except g_*j*_ and *δ*_*j*_, respectively, and **σ** ^2^ = (*σ*_1_^2^, *σ*_2_^2^). Then the conditional distribution for *δ*_*j*_ can be written as:

17 $$\begin{array}{cc}f({\delta }_{j}|{\bf{y}},{\bf{u}},{{\bf{g}}}_{-j},{{\boldsymbol{\delta }}}_{-j},{{\boldsymbol{\sigma }}}^{2},{{\sigma }_{e}}^{2},\gamma ) & \propto \,f({\bf{y}}|{\delta }_{j},{\bf{u}},{{\bf{g}}}_{-j},{{\boldsymbol{\delta }}}_{-j},{{\boldsymbol{\sigma }}}^{2},{{\sigma }_{e}}^{2},\gamma )p({\delta }_{j}|\gamma )\\  & \propto \,f({{\bf{y}}}_{-j}|{\delta }_{j},{{\sigma }_{j}}^{2},{{\sigma }_{e}}^{2})p({\delta }_{j}|\gamma )\end{array},$$

where ${{\bf{y}}}_{-j}={\bf{y}}-{\bf{X}}{\bf{u}}-\sum _{k\ne j}{{\bf{B}}}_{k}{g}_{k}={{\bf{B}}}_{j}{g}_{j}+{\bf{e}}$, as in equation (4). Thus, $f({{\bf{y}}}_{-j}|{\delta }_{j},{{\sigma }_{j}}^{2},{{\sigma }_{e}}^{2})p({\delta }_{j}|\gamma )$ can be represented as:

18 $$f({{\bf{y}}}_{-j}|{\delta }_{j},{{\sigma }_{j}}^{2},{{\sigma }_{e}}^{2})p({\delta }_{j}|\gamma )=\{\begin{array}{c}f({{\bf{y}}}_{-j}|{{\sigma }_{1}}^{2},{{\sigma }_{e}}^{2})\gamma \quad \quad \,\,\,\,{\rm{when}}\,{\delta }_{j}=1\\ f({{\bf{y}}}_{-j}|{{\sigma }_{2}}^{2},{{\sigma }_{e}}^{2})(1-\gamma )\quad {\rm{when}}\,{\delta }_{j}=0\end{array}.$$

As $f({\delta }_{j}=1|{\bf{y}},{\bf{u}},{{\bf{g}}}_{-j},{{\boldsymbol{\delta }}}_{-j},{{\boldsymbol{\sigma }}}^{2},{{\sigma }_{e}}^{2},\gamma )+f({\delta }_{j}=0|{\bf{y}},{\bf{u}},{{\bf{g}}}_{-j},{{\boldsymbol{\delta }}}_{-j},{{\boldsymbol{\sigma }}}^{2},{{\sigma }_{e}}^{2},\gamma )=1$, $f({\delta }_{j}=1|{\bf{y}},{\bf{u}},{{\bf{g}}}_{-j},{{\boldsymbol{\delta }}}_{-j},$ ${\sigma }^{2},{\sigma }_{e}^{2},\gamma )$ can be sampled from:

19 $$\begin{array}{c}f({\delta }_{j}=1|{\bf{y}},{\bf{u}},{{\bf{g}}}_{-j},{{\boldsymbol{\delta }}}_{-j},{{\boldsymbol{\sigma }}}^{2},{{\sigma }_{e}}^{2},\gamma )\\ =\frac{f({\delta }_{j}=1|{\bf{y}},{\bf{u}},{{\bf{g}}}_{-j},{{\boldsymbol{\delta }}}_{-j},{{\boldsymbol{\sigma }}}^{2},{{\sigma }_{e}}^{2},\gamma )}{f({\delta }_{j}=1|{\bf{y}},{\bf{u}},{{\bf{g}}}_{-j},{{\boldsymbol{\delta }}}_{-j},{{\boldsymbol{\sigma }}}^{2},{{\sigma }_{e}}^{2},\gamma )+f({\delta }_{j}=0|{\bf{y}},{\bf{u}},{{\bf{g}}}_{-j},{{\boldsymbol{\delta }}}_{-j},{{\boldsymbol{\sigma }}}^{2},{{\sigma }_{e}}^{2},\gamma )}\\ =\frac{f({{\bf{y}}}_{-j}|{{\sigma }_{1}}^{2},{{\sigma }_{e}}^{2})\gamma }{f({{\bf{y}}}_{-j}|{{\sigma }_{1}}^{2},{{\sigma }_{e}}^{2})\gamma +f({{\bf{y}}}_{-j}|{{\sigma }_{2}}^{2},{{\sigma }_{e}}^{2})(1-\gamma )}\\ =\frac{1}{1+\frac{f({{\bf{y}}}_{-j}|{{\sigma }_{2}}^{2},{{\sigma }_{e}}^{2})(1-\gamma )}{f({{\bf{y}}}_{-j}|{{\sigma }_{1}}^{2},{{\sigma }_{e}}^{2})\gamma }}.\end{array}$$

Note that *f*(**y**_*−j*_|*σ*_*j*_^2^, *σ*_*e*_^2^) is a multivariate density, the case of which is similar to that in FMixP. An efficient way is to use the product of univariate distributions of **B**_*j*_′**y**_*−j*_ instead of the distribution of **y**_*−j*_^13,14^. The *f*(**B**_*j*_′**y**_*−j*_|${\sigma }_{j}^{2}$, ${\sigma }_{e}^{2}$) has zero mean and variance (**B**_*j*_′**B**_*j*_)^2^ ${\sigma }_{j}^{2}$ + **B**_*j*_′**B**_*j*_ ${\sigma }_{e}^{2}$. Thus, the equation (19) can be written as:

20 $$\begin{array}{lll} &  & f({\delta }_{j}=1|{\bf{y}},{\bf{u}},{{\bf{g}}}_{-j},{{\boldsymbol{\delta }}}_{-j},{{\boldsymbol{\sigma }}}^{2},{{\sigma }_{e}}^{2},\gamma )\\  & = & \frac{1}{1+\frac{f({{\bf{B}}}_{j}^{^{\prime} }{{\bf{y}}}_{-j}|{\sigma }_{2}^{2},{\sigma }_{e}^{2})(1-\gamma )}{f({{{\bf{B}}}_{j}}^{^{\prime} }{{\bf{y}}}_{-j}|{\sigma }_{1}^{2},{\sigma }_{e}^{2})\gamma }}\\  & = & \frac{1}{1+\exp [0.5\,\mathrm{log}({V}_{1})-0.5\,\mathrm{log}({V}_{2})+\frac{0.5{({{\bf{B}}}_{j}^{^{\prime} }{{\bf{y}}}_{-j})}^{2}}{{V}_{1}}-\frac{0.5{({{\bf{B}}}_{j}^{^{\prime} }{{\bf{y}}}_{-j})}^{2}}{{V}_{2}}+\,\mathrm{log}(1-\gamma )-\,\mathrm{log}(\gamma )]},\end{array}$$

where *V*_1_ = (**B**_*j*_′**B**_*j*_)^2^ *σ*_1_^2^ + **B**_*j*_′**B**_*j*_ *σ*_*e*_^2^ and *V*_2_ = (**B**_*j*_′**B**_*j*_)^2^ ${\sigma }_{2}^{2}$ + **B**_*j*_′**B**_*j*_ ${\sigma }_{e}^{2}$. After the *δ*_*j*_ has been updated, *g*_*j*_ is sampled as:

21 $$f({g}_{j}|{\delta }_{j},{\bf{y}},{\bf{u}},{{\bf{g}}}_{-j},{{\boldsymbol{\delta }}}_{-j},{{\boldsymbol{\sigma }}}^{2},{{\sigma }_{e}}^{2},\gamma ) \sim \{\begin{array}{c}N(\frac{{{\bf{B}}{\boldsymbol{^{\prime} }}}_{j}{{\bf{y}}}_{-j}}{{{\bf{B}}{\boldsymbol{^{\prime} }}}_{j}{{\bf{B}}}_{j}+{{\sigma }_{e}}^{2}/{{\sigma }_{1}}^{2}},\frac{{{\sigma }_{e}}^{2}}{{\bf{B}}{^{\prime} }_{j}{{\bf{B}}}_{j}+{{\sigma }_{e}}^{2}/{{\sigma }_{1}}^{2}})\quad if\,{\delta }_{j}=1\\ N(\frac{{{\bf{B}}}_{j}^{^{\prime} }{{\bf{y}}}_{-j}}{{\bf{B}}{^{\prime} }_{j}{{\bf{B}}}_{j}+{\sigma }_{e}^{2}/{\sigma }_{2}^{2}},\frac{{{\sigma }_{e}}^{2}}{{\bf{B}}{^{\prime} }_{j}{{\bf{B}}}_{j}+{\sigma }_{e}^{2}/{\sigma }_{2}^{2}})\quad if\,{\delta }_{j}=0\end{array}.$$

As the *σ*_1_^2^ appears only in its own prior and the normal distribution of *g*_*j*_ with *δ*_*j*_ = 1, the posterior distribution of *σ*_1_^2^ can be derived as:

22 $$\begin{array}{lll}f({{\sigma }_{1}}^{2}|{\bf{y}},{\bf{u}},{\bf{g}},{\boldsymbol{\delta }},{{\sigma }_{2}}^{2},{{\sigma }_{e}}^{2},\gamma ) & \propto  & f({{\sigma }_{1}}^{2}){\prod }_{{\delta }_{j=1}}f({g}_{j}|{{\sigma }_{1}}^{2})\\  & \propto  & {({{\sigma }_{1}}^{2})}^{-\frac{k+v+2}{2}}\exp (-\frac{v{{s}_{1}}^{2}+{\sum }_{{\delta }_{j=1}}{{g}_{j}}^{2}}{2{{\sigma }_{1}}^{2}})\\  &  \sim  & {\chi }^{-2}(k+v,\frac{v{{s}_{1}}^{2}+{\sum }_{{\delta }_{j=1}}{{g}_{j}}^{2}}{k+v})\end{array},$$

where *k* is the number of SNP loci with *δ*_*j*_ = 1. Similarly, the posterior distribution of ${{\sigma }_{2}}^{2}$ follows the inverse-chi-squared distribution ${\chi }^{-2}(m+v,\frac{v{{s}_{2}}^{2}+{\sum }_{{\delta }_{j=0}}{{g}_{j}}^{2}}{m+v})$, where *m* is the number of SNP loci with *δ*_*j*_ = 0.

The starting value of *γ* was set to 0.5, and the posterior probability is drawn from the Beta(*k* + 1, *m* + 1):

23 $$\begin{array}{lll}f(\gamma |{\bf{y}},{\bf{u}},{\bf{g}},{\boldsymbol{\delta }},{{\boldsymbol{\sigma }}}^{2},{{\sigma }_{e}}^{2}) & \propto  & f(\gamma )f({\boldsymbol{\delta }}|\gamma )\\  & \propto  & {\gamma }^{k}{(1-\gamma )}^{m}\end{array}.$$

Note that if the sampling value of *γ* is larger than 0.5, we can switch the labels of the variance ${{\sigma }_{1}}^{2}$ and ${{\sigma }_{2}}^{2}$, and set value of *γ* to 1 − *γ*. The posterior distributions of fixed effect **u** and residual variance ${{\sigma }_{e}}^{2}$ are the same as BayesA, which has been described in many studies^1,13,15^.

### Genomic prediction by other approaches

Two other Bayesian methods, BayesA^1^ and BayesCπ^3^, were used for comparison with MixP. The prior distribution of variances of SNP effects in BayesA follows an inverse-chi-squared distribution, i.e., *σ*_*j*_^2^ ~ *χ* ^−2^(*v*, *s* ^2^)^1,11^. In BayesCπ, SNPs with non-zero effects have a common variance that also follows an inverse-chi-squared distribution^3^. The degree of freedom (*v*) of the inverse-chi-squared distribution was set to 5.0. As the SNP genotypes had been standardised, parameter *s* ^2^ was set without ∑2*p*_*j*_(1 − *p*_*j*_) in the denominator, which was different from the formula derived by Habier *et al*.^3^ and Gianola *et al*.^16^. In this study, *s* ^2^ = [(*v* − 2)*V*_*g*_]/(*vM*) in BayesA and *s* ^2^ = [(*v* − 2)*V*_*g*_]/(*πvM*) in BayesCπ, where *π* represents the probability of a SNP with a non-zero effect and is estimated by the MCMC algorithm. *V*_*g*_ is total additive genetic variance which is estimated using the R-package “EMMREML” (Version 3.1) that is one of packages^17–22^ used to estimate genetic parameters. Before *V*_*g*_ estimation, a genomic relationship matrix (**G** matrix) was calculated using the formula^2^: ${\bf{G}}=\frac{({\bf{B}}{\boldsymbol{-}}{\bf{P}}{\boldsymbol{)}}{\boldsymbol{(}}{\bf{B}}{\boldsymbol{-}}{\bf{P}})^{\prime} }{2\sum {p}_{j}(1-{p}_{j})}$, where the *j*th column of P is a vector of the frequency of allele ‘a’ at the *j*th locus, i.e., ${{\bf{P}}}_{j}=({p}_{j},{p}_{j},\mathrm{..}.,{p}_{j})^{\prime}$. Gibbs sampling was run for 20000 cycles, and the first 10000 cycles were discarded as burn in.

### Simulated data

Both the simulated and real data were used to compare the predictive results of various statistical methods. The simulated data had been distributed to the participants of the QTLMAS XII workshop. The data was described in detail by Lund *et al*.^23^ and a summary is given as follows. Through a simulation of a historic population of 50 generations, 4665 and 1200 individuals were simulated in the training and testing data sets, respectively. Six-thousand biallelic SNP loci were evenly spaced on 6 Morgan chromosomes, and 5,726 SNPs with minor allele frequencies (MAF) ≥0.05 were used for research. Forty-eight QTL loci were simulated, and the effects were sampled from a gamma distribution with a scale parameter 5.4 and a shape parameter of 0.42. The residual values were sampled to obtain a heritability value of 0.3 for the trait.

### Real data on large yellow croaker

The experimental materials were large yellow croaker (*Larimichthys crocea*), which is one of the most commercially important marine fish species in southeast China and Eastern Asia^24^. All fish were reared in a breeding nucleus farm named ‘Jinling Aquaculture Science and Technology Co. Ltd.’ in Ningde City, Fujian Province, P.R. China. In total, 30 males and 30 females were mated randomly in a pool, and a total of 500 progenies (237 males and 263 females) were randomly selected and measured in the experiment. The trial was carried out in the Key Laboratory of Healthy Mariculture for the East China Sea when the fish were two years old. Four quantitative traits, body weight, body length, body height and the length/height ratio, were selected to perform genomic prediction. Growth rate and body shape (customers prefer purchasing fish with slender bodies) are the important traits for large yellow croaker, so these four traits were selected for research. The parameters of the four traits are shown in Table 4.

**Table 4:** Statistical results of the phenotypic data for four quantitative traits in large yellow croaker.

Trait	Male		Female		Heritability^b^
	Number	Mean^a^ ± SE	Number	Mean^a^±SE	(Mean±SE)
Body weight	237	202.22 ± 5.01	263	247.41 ± 6.16	0.61 ± 0.11
Body length	237	227.19 ± 1.64	263	234.85 ± 1.79	0.59 ± 0.10
Body height	237	62.03 ± 0.53	263	66.61 ± 0.59	0.52 ± 0.11
Length/height	237	3.68 ± 0.01	263	3.54 ± 0.01	0.32 ± 0.10

^a^The units are gram (g) for body weight, and millimetre (mm) for body length and body height.

### Next generation sequencing and genotyping

Fin samples from 500 individuals were collected for genotyping. The Genotyping-By-Sequencing (GBS) method was used to construct the libraries for next generation sequencing (NGS). Genomic DNA was incubated at 37 °C with *Eco*RI and *Nla*III, CutSmart™ buffer and MilliQ water. Digestion reactions were heat-inactivated at 65 °C for 20 minutes and the reaction system was held at 8 °C. The digested DNA was ligated to adapter sequences with CutSmart™ buffer, ATP, T4 DNA ligase, adapter mix and MilliQ water at 16 °C. The restriction-ligation reaction was also heat-inactivated at 65 °C for 20 minutes and the reaction system was held at 8 °C afterward. The PCR reaction was performed using diluted restriction-ligation samples, dNTP, Taq DNA polymerase (NEB) and IlluminaF and indexing primers. Fragments that were 200~300 bp in size were isolated using a Gel Extraction Kit (Qiagen). Then, pair-end sequencing was performed using an Illumina high-throughput sequencing platform. The raw sequencing reads were quality checked by FastQC^25^. The high-quality, filtered reads were mapped to the large yellow croaker reference genome sequence by BWA version 0.7.10^26^. The alignment files were then sorted and the duplicates marked by Picard (http://picard.sourceforge.net). Then, the GATK package^27^ was applied for SNP calling. As a result, 29,748 SNPs with a missing rate ≤20%, a MAF (minor allele frequency) ≥0.05 and genotypes in Hardy-Weinberg equilibrium were selected for further analysis. Beagle Version 3.3.2 software was used to impute the missing SNPs^28^.

### Cross-validation

Genomic prediction by a replicated training-testing method was used to evaluate the predictive results of the real data. Cross-validation of 10 replicates was performed. All 500 individuals were randomly and evenly divided into 10 groups of 50 individuals each. In each replicate, one of the groups was selected as the testing data set while the remaining nine groups were used as the training data set. To observe the relationship between the predictive results of FMixP with *γ*, we varied the value of *γ* from 0.01 to 0.5 (50 levels were used with 0.01 as a step size).

### Predictive accuracy and predictive ability

In the simulation, the correlation coefficient between true genetic values and predicted genetic values, *r*_(TBV_,_GEBV)_ was used to measure the predictive accuracy^1^, where **GEBV** = **Bĝ**, **ĝ** is the vector of estimated SNP effects and **B** is the SNP genotypes; and an individual true breeding value (TBV) can be obtained by summing up all simulated QTL effects. We give a brief explanation below. If an individual GEBV is close to its TBV, the predictive accuracy is high. But if one aims to assess the predictive accuracies of a set of GEBV, one can use *r*_(TBV_,_GEBV)_, and higher *r*_(TBV_,_GEBV)_ suggests higher predictive accuracy.

In the real data analysis, because the true breeding values are unknown, we used the predictive ability to measure the predictive accuracy, which is described as the correlation coefficient between **GEBV**, and the phenotypes adjusted for the covariates (**y** − **X û **, where only genetic and residual effects are left), *r*_(**y***−***Xû**_, _**GEBV**)_^29^. The higher correlation between them is, the higher genetic variance captured by the genetic SNPs is, leading to higher predictive ability.

All 500 individuals were added to the prediction model to estimate the computation time for various Bayesian methods. All of the calculation processes (except the REML process) were implemented in Fortran90 codes and run on the computer server of Jimei University.

### Availability of data

Raw DNA sequencing reads were deposited in NCBI with the project accession PRJNA309464 and SRA accession SRR3114179.
